# Dynamic Radiographs in Assessing Stability of Cervical Spine Fractures: A Multicentre Study

**DOI:** 10.5435/JAAOSGlobal-D-22-00067

**Published:** 2022-10-21

**Authors:** Ruben Patrick Thumbadoo, Jan Herzog, Niv Bhamber, Cristina Lupu, Kenny Kwan, Andrew Clarke, Michael Hutton, Jason Bernard, Timothy Bishop, Darren F. Lui

**Affiliations:** From the St George's University NHS Foundation Trust, London, United Kingdom (Thumbadoo, Herzog, Bhamber, Lupu, Bernard, Bishop, and Lui); the Queen Mary Hospital, Hong Kong, Hong Kong (Kwan); and the Royal Devon and Exeter Hospital, Exeter, United Kingdom (Clarke and Hutton).

## Abstract

In the management of a trauma patient with cervical spine injury, the need for accurate diagnostic imaging is key to ensure correct management. Different classification systems have been developed including the Subaxial Injury Classification (SLIC) system and AO cervical spine fracture classification. Through a multicentre study, we have identified a group of cases where the use of CT alone to classify fractures by either SLIC or AO score may be deficient and the use of dynamic cervical spine radiographs could help identify instability.

**Methods:**

Three level 1 trauma centers retrospectively reviewed patients with cervical spine injuries. Cervical spine radiographs (AP and lateral) were undertaken in collar, in all patients with suspected cervical spine injury within 2 weeks, followed by reanalysis of scoring systems.

**Results:**

Eleven cases were identified in total, and 72% were male with a mean age of 65 years, with approximately 54% being older than 70 years. All patients reported their pain as severe using the Visual Analogue Scale scale. The predynamic radiograph mean SLIC score was 0.73, which is in contrast to the postdynamic radiograph mean SLIC score of 6. The statistical significance (*P* = 0.004) was found using the Wilcoxon signed-rank test.

**Conclusion:**

Supine imaging eliminates the gravitational loads normally exerted on the c-spine. The cases show assumed cervical stability based on CT, but dynamic c-spine radiographs subsequently demonstrated instability. Therefore, we suggest a combination of SLIC and AO classification using radiologic imaging to classify fracture and correlate clinical symptoms with persistent neck pain, which warrants a Miami-J collar and dynamic c-spine radiograph to assess stability with re-evaluation of scoring.

According to the 2008 study by Milby et al,^1^ the overall prevalence of cervical spine (c-spine) injury in all trauma patients was 3.7%. In the management of a trauma patient, especially with potential c-spine injuries, the need for accurate diagnostic imaging is paramount to ensure complications such as paralysis or even death are avoided.^2^ These complications generally occurred in unstable c-spine trauma, which can arise in around 40% of spinal trauma cases. Hyperflexion of the c-spine can cause instability because of facet joint dislocation, although other problems such as posterior ligament injury and pure transosseous lesions could also be causes. Facet joint dislocations are categorized by the amount of anterior subluxation at the level of injury and factors such as size and type of fracture. A unilateral facet dislocation has greater stability and exhibits only 25% anterior movement, in contrast to a bilateral facet dislocation, which shows 50% anterior movement, but instability is not always obviously identifiable.

NICE guidelines and the Advanced Trauma Life Support (ATLS) protocol guides decision making in the UK trauma setting. The NICE guidelines suggest that c-spine pain requires a c-spine CT, with an obviously unstable fracture treated by surgical intervention. A stable fracture or facet joint dislocation is usually treated with a collar, but it is difficult to assess stability.

Different classification systems have been developed such as the Subaxial Injury Classification (SLIC) system. The SLICS score is a classification determined by the Spine Trauma Study Group that can be used to guide management based on neurologic symptoms, morphology, and discoligament complex integrity based on radiologic evidence.^3^ For a severity score of less than 4, conservative management has been suggested. For a score of ≥ 5, surgical treatment has been suggested. A score of 4 may be treated in either way depending on surgeon preference. The current decision-making protocol is outlined in Table 1.

**Table 1:** Current Trauma Protocol and Treatment

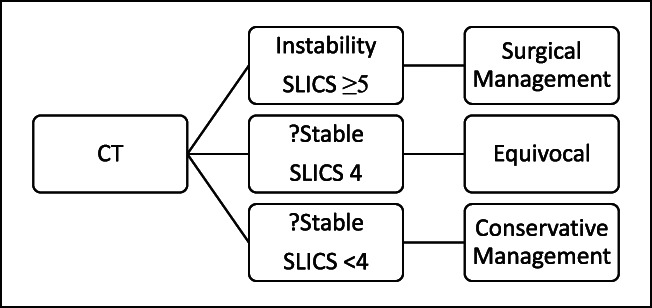

In 2015, the AO Spine Knowledge Forum developed a user-friendly classification system for subaxial cervical spine injuries.^4^ The classification system describes injuries based on four criteria: morphology of the injury, facet injury, neurologic status, and any case-specific modifiers. Three injury morphology types are similar to the thoracolumbar system: compression injuries (A), tension band injuries (B), and translational injuries (C), with additional descriptions for facet injuries (F), as well as patient-specific modifiers (M) and neurologic status. These letters would then be used in the final nomenclature of the fracture. However, it does not guide surgery or prognosticate. Through a multicenter study, we have identified a group of cases where the use of CT alone to classify fractures by either SLIC or AO score may be deficient, and the use of erect or dynamic c-spine radiographs can help identify instability.

## Methods

Three centers reviewed patients with cervical spine injuries, which included Queen Mary Hospital in Hong Kong and Royal Devon and Exeter Hospital and St George's Hospital in London, two NHS foundation trusts in the United Kingdom. These hospitals were chosen because they routinely use erect c-spine or dynamic c-spine radiographs in patients who sustain cervical spine fractures. Because this audit did not affect daily clinical practice, no formal approval was requested. Using the NHS Research authority tool, ethics approval deemed unnecessary according to national regulations. However, this study was conducted according to the ethical principles stated in the Declaration of Helsinki. For this type of study, formal consent to participate is not required.

**Figure 1:**
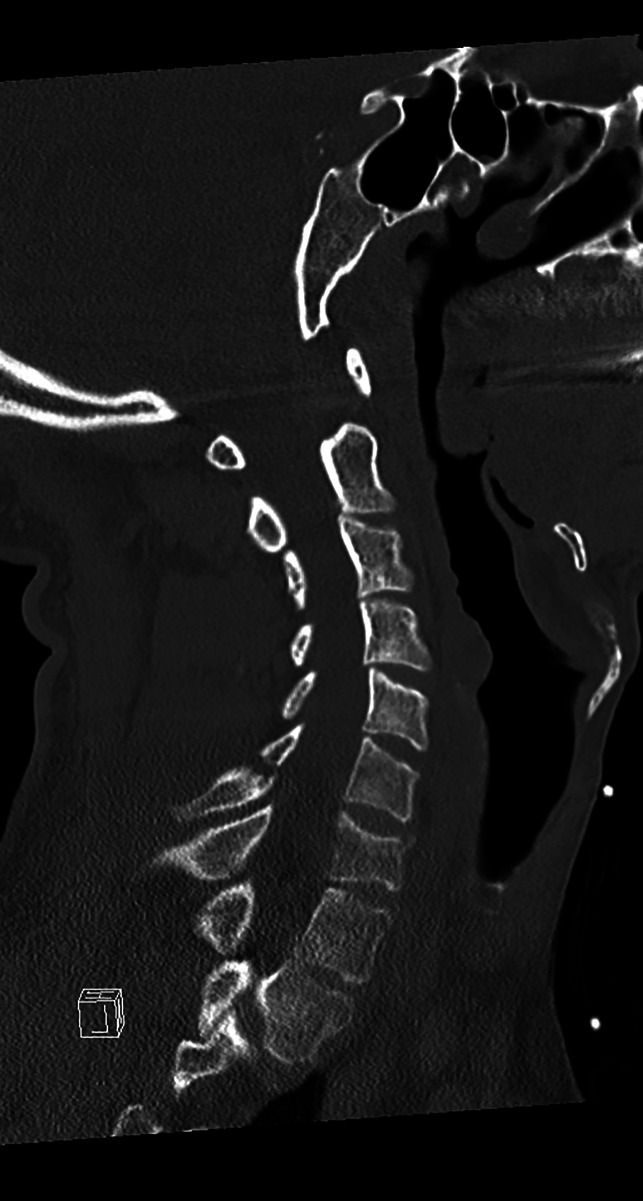
CT scan showing C6 spinous process fracture seen on CT.

**Figure 2:**
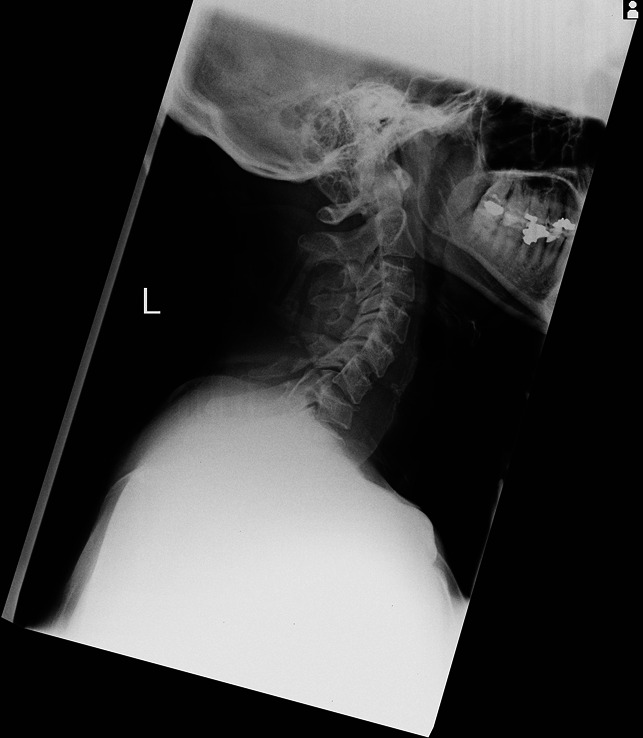
Erect c-spine radiograph showing instability.

**Figure 3:**
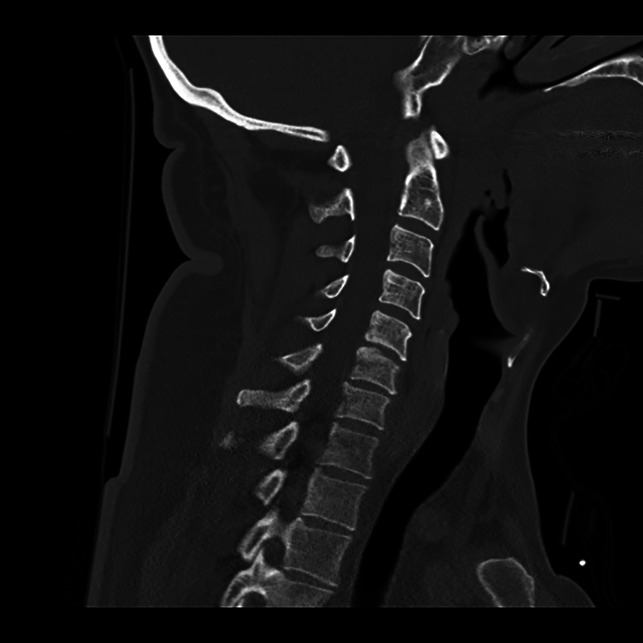
Radiograph showing undisplaced right C6 facet fracture. Potential for instability AO SLIC (F2).

**Figure 4:**
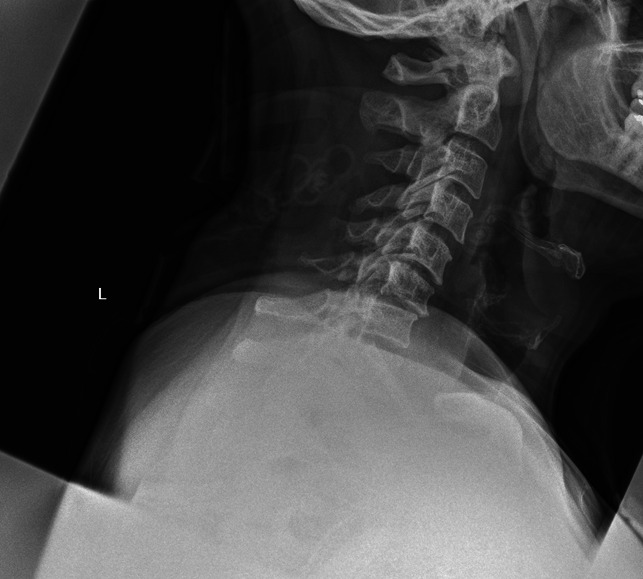
Erect c-spine radiograph conducted on arrival to clinic showing instability.

**Figure 5:**
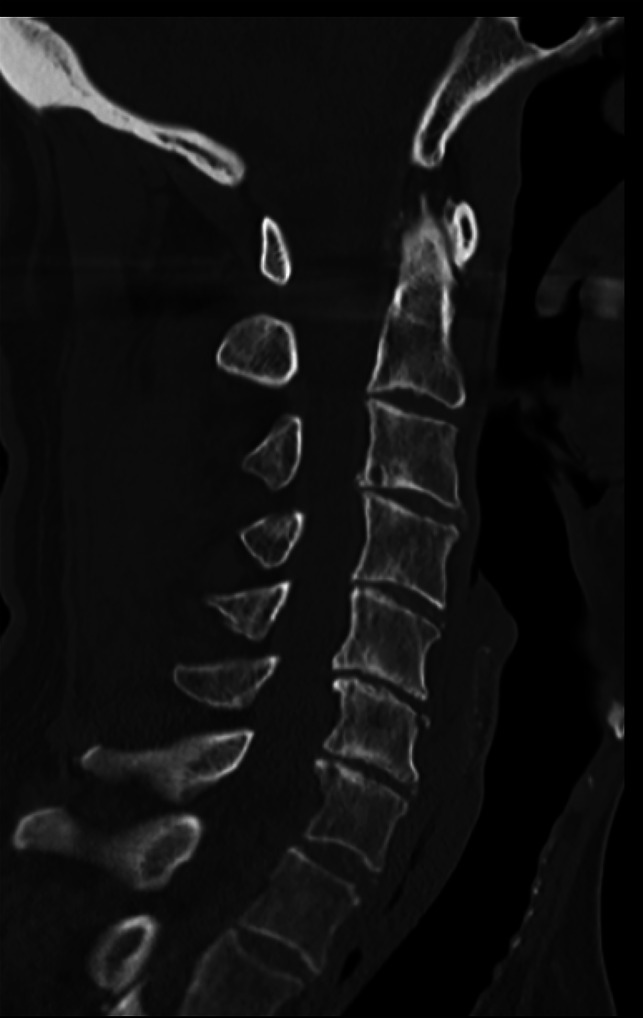
HK CT scan showing degenerative changes but no obvious fracture.

**Figure 6:**
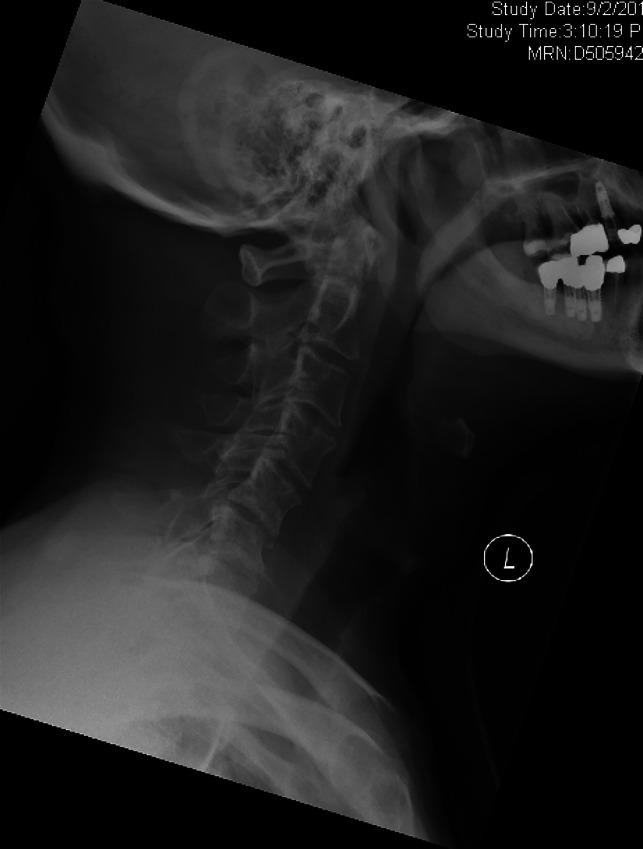
Radiograph showing instability in the HK case.

**Figure 7:**
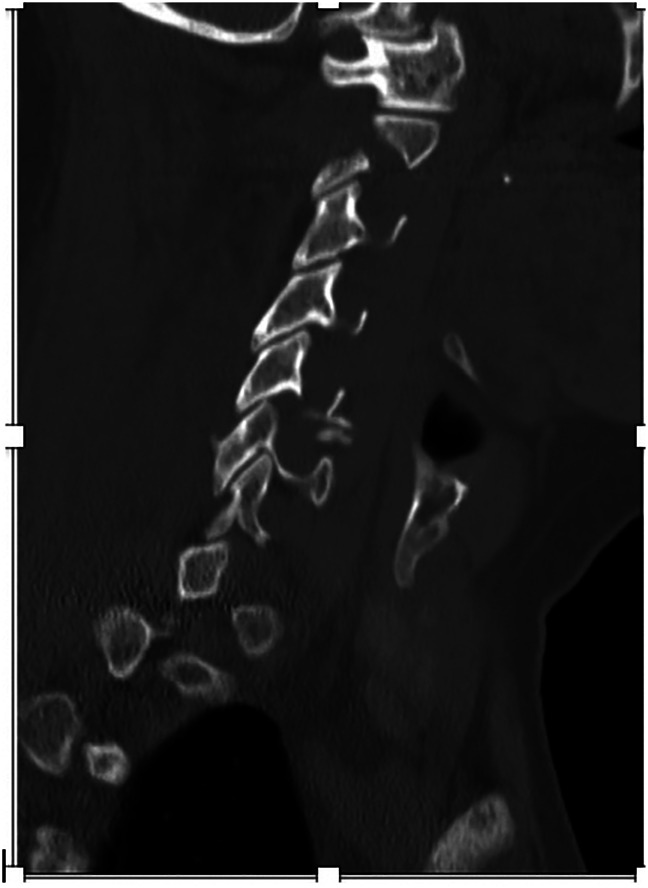
Exeter CT—sagittal CT scan, made with the patient supine, reported as showing widespread degenerative changes.

**Figure 8:**
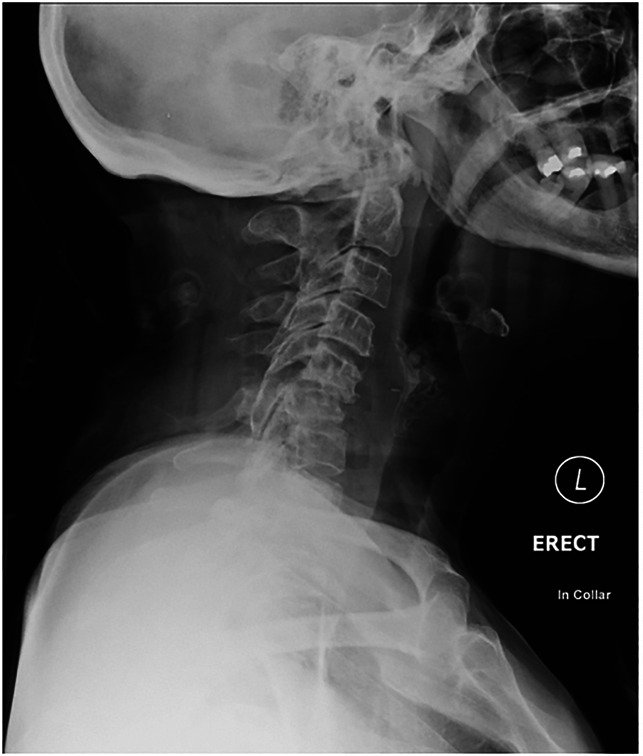
Exeter radiograph—erect lateral cervical spine radiograph demonstrating C5-C6 fracture subluxation.

This was a nonconsecutive series of patients identified where cases were retrospectively identified and included in the cohort. Our orthopaedic practice is a trauma CT series including the head, whole spine, and pelvis with clinical evaluation and application of the AO cervical classification and SLICS score. Erect or dynamic c-spine radiographs (AP and lateral) are undertaken while wearing a collar, in all patients with suspected c-spine injury before discharge or within 2 weeks at their follow-up outpatient appointment, followed by reanalysis of the AO classification and SLIC. Examples of cases comparing CT scans and c-spine radiographs are depicted in Figures 1 to 8.

## Results

Eleven cases were identified in total, and 72% were male with a mean age of 65 years, with approximately 54% being older than 70 years, as presented in Table 2. All patients reported their pain as severe using the Visual Analogue Scale scale,^5^ meaning 8 and more of a possible maximum score of 10. We have used the SLIC to score the cases of both predynamic and postdynamic/erect c-spine radiographs presented in Table 3. The predynamic radiograph mean SLIC score was 0.73, which is in contrast to the postdynamic radiograph mean SLIC score of 6. Using the Wilcoxon signed-rank test, the *P*-value was 0.004. This statistical significance rejects the null hypothesis that the samples follow the same distribution and accepts the alternative hypothesis that the samples are different because of the use of dynamic/erect cervical spine radiographs. Table 4 lists the AO classification of the cases, showing the change in scoring between image modalities.

**Table 2:** Demographics of Patients

Cases	Age	Sex	MOI	Pain Score
1	28	Male	Crushed under the wheel of a lorry	Severe
2	50	Male	2.5-m fall	Severe
3	89	Male	Fall downstairs at home	Severe
4	47	Male	Driver—RTC at 80 MPH	Severe
5	73	Male	RTC	Severe
6	72	Male	Fall downstairs at home	Severe
7	75	Male	Driver—RTC	Severe
8	61	Female	Fell from a horse	Severe
9	61	Female	Fell backward	Severe
10	75	Male	Fell backward	Severe
11	87	Female	Passenger—low-speed RTC	Severe

DLC = discoligament complex integrity, HK = Hong Kong, MOI = mechanism of injury, RTC = road traffic collision

**Table 3:** Subaxial Injury Classification System (SLIC) Scoring for the 11 Cases Showing That Potential Unstable Fractures Can Be Missed Without Erect C-Spine Radiographs

	Morphology		DLC		Neuro status		Total	
	Pre	Post	Pre	Post	Pre	Post	Pre	Post
1	0	4	0	2	0	0	0	6
2	0	4	0	0	1	1	1	5
3	3	4	0	2	0	0	3	6
4	3	3	0	2	0	1	3	6
5	0	4	0	2	0	0	0	6
6	0	4	0	2	0	0	0	6
7	0	4	0	2	0	0	0	6
8	0	4	0	2	0	1	0	7
9	0	4	1	2	0	0	1	6
10	0	4	0	2	0	0	0	6
11	0	4	0	2	0	0	0	6
Mean							0.73	6
*P* value	0.004							

**Table 4:** AO Scoring for the 11 Cases Showing That Potential Unstable Fractures Can Be Missed Without Erect C-Spine Radiographs

	CT	Erect/Dynamic Radiograph
1	A0, F1	A0, F4
2	C6 F1	C6 BL F4; C7 BL F4
3	F1	F4
4	F2	C, F4
5	B2, F1	C, B2, F4
6	F2	F4
7	No injury	F4
8	F2	F4
9	F1	F4
10	A1	A1, C
11	No injury	F4

## Discussion

White and Panjabi^6^ described c-spine instability as the loss of the spine's ability to maintain its patterns of displacement under physiological loads, so there are no initial or additional neurologic deficit, no major deformity, and no incapacitating pain. The SLIC score is a classification determined by the Spine Trauma Study Group that can be used to guide management based on clinical symptoms and radiologic evidence.^3^ We suggest that a patient who scores 4 or less undergoes additional c-spine radiograph, either dynamic or erect, to assess stability because unstable fractures could otherwise be missed in the crucial immediate stages for lower-risk patients.

The NICE guidelines regarding cervical injury recommend CT scan for adults if indicated by the Canadian C-Spine Rule (CCR).^7^ If any neurologic pathology or deficiency is observed, MRI is also recommended. The CCR uses three clinical questions to assess the need for c-spine imaging. The first assesses age, mechanism of injury, and neurologic deficiency to categorize high-risk patients who must undergo imaging. The second assesses low-risk characteristics, which may undergo a safe assessment of active range of motion. Finally, the three questions assess the ability of the patient to actively rotate their neck 45° to the left and to the right, regardless of pain, which would not require imaging. The CCR is used to decide the need for imaging, but not the type of imaging. At St George's Hospital, all major trauma patients undergo head, abdominal, and pelvic CTs when stable.^8^ The literature shows that CT markedly outperforms radiographs as a screening tool for the identification of cervical injury in high-risk patients, but there is not enough evidence to suggest its use in low-risk patients.^9^

The use of erect c-spine or dynamic (flexion-extension) radiographs is not part of the NICE guidelines, but the literature suggests the need for additional investigation into their use as an adjunct,^10,11^ a viewpoint we support. CT has replaced dynamic radiographs, although theoretically, only dynamic radiography can diagnose instability of the c-spine.^12^ CT can propose instability only by suggesting ligamentous injury, although this may be more difficult when subtle soft-tissue changes are present in comparison with fractures. However, image quality can often be dubious,^13^ visualization of the entire c-spine is often missing, and adverse neurologic events due to movement may occur.^14^ The diagnostic value of dynamic imaging in addition to CT or MRI is minimal.^12^ Insko et al^15^ suggested that neck pain and spasm may limit the ability to flex and extend the c-spine and that dynamic imaging may yield false-negative results; however, they excluded any cases of dynamic imaging obtained beyond 12 hours after the initial evaluation.

Previously, the American College of Radiologists (ACR) recommended using radiographs in an erect position “since it better demonstrates instability.”^16^ However, the current ACR guidelines suggest that radiography is of limited use, CT is the first line for determining cervical injury and stability, and radiographs should be reserved for use with CT scans affected by movement artifact. However, it has previously been commented that similar to all major joints, c-spine injuries should be radiographed under load if the initial imaging does not reveal an abnormality, but there are signs and symptoms.^17^ Supine imaging eliminates the gravitational loads normally exerted on the c-spine. During erect radiography, the muscles and ligaments are under strain, and therefore, instability due to ligament damage can be demonstrated.

By contrast, spinal stability in the thoracic and lumbar spine was classified by the Spine Trauma Study Group in the 2005 Thoracolumbar Injury Classification and Severity Score (TLICS).^18^ One measure of the score is the integrity of the posterior ligament complex (PLC), on a three-point scale, with assessment based on plain radiographs, CT scans, and MRI.^18^ However, in the more recently updated AO Spine Thoracolumbar Spine Injury Classification System by Vaccaro et al,^19^ the value of PLC injury has been reduced to a one-point modifier. This change is due to the difficulty in reliably identifying PLC injury on imaging. Although we are aware this classification is for the thoracolumbar spine, the difficulty in assessment of ligamentous injury on imaging is equally valid in the cervical spine.

In the cases within our series however, the fractures were shown to be stable by CT only on further imaging was instability of the fracture demonstrated. Erect c-spine radiograph and MRI both showed that the fracture was unstable, hence necessitating the need for reduction and fixation of the fractures. MRI has greater sensitivity to showing spinal instability than a CT scan.^10,20^ Brandstein et al^11^ demonstrated a relatively small number of unstable fractures missed on CT; however, they also highlighted the benefits of erect lateral radiograph, which showed instability in all cases previously unseen on CT and MRI.

The use weighty‐bearing imaging to illustrate instability has previously been utilised and been standard until superseded by CT and MRI. However, MRI can only suggest stability and cannot prove it.^10^ There is a lack of widespread availability of MRI along with high costs associated compared with the cheap and easily available radiograph.

The abovementioned cases show the importance of simple dynamic or erect radiograph imaging in demonstrating instability, in conjunction with the SLICS classification as a marker of stability. An updated protocol including the use of dynamic/erect radiograph is presented in Table 5.

**Table 5:** Potential Protocol Including Dynamic/Erect c-Spine Radiographs

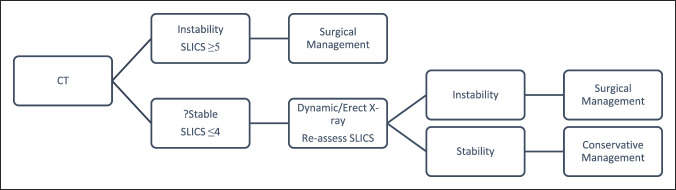

This study also has limitations. Although the sample size is small and these are nonsequential cases that have been analyzed retrospectively, it is always important to have a level of clinical suspicion even if cervical spine fracture stability is shown by supine imaging and cervical immobilization started. Other limitations include the wide time frame to collect the sample size seen. This is because of the complex presentation, among only a small number of hospitals. There continues to be a role of MRI in the obtunded patient or too unwell to have a departmental radiograph since an erect c-spine radiograph can only be done in a well patient.

## Conclusion

As per the ACR, we suggest that erect C-spine radiographs are used.^12^ It is easier to conduct than a dynamic view and can be done in the cervical collar. It may obviate the need for MRI. We think that the use of erect c-spine radiographs as an adjunct can help delineate cervical instability. This could alter the AO classification and SLIC score. Therefore, we suggest a useful modification to the SLIC algorithm with the use of erect C-spine radiographs, particularly if pain is persistent, to better illustrate instability and therefore guide the correct care at the right time.
